# Supplementary material for: Asymmetric Dimethylaminohydrolase Gene Polymorphisms Associated with Preeclampsia Comorbid with HIV Infection in Pregnant Women of African Ancestry
Source: Int J Mol Sci. 2025 Apr 1;26(7):3271. doi: 10.3390/ijms26073271 (PMC11989882; doi:10.3390/ijms26073271)
Supplement: Supplementary file 1 [file ijms-26-03271-s001.zip › ijms-3364767-supplementary.pdf]

Supplementary Table S1: Genotype and allele frequency distribution (%) of *ADMA* gene polymorphisms (*rs669173*, *rs7521189*, *rs805305*, *rs3131383*) across pregnancy types, stratified by HIV status.

SNP ID		Normotensive		Preeclamptic								Pregnancy Type		HIV Status	
**rs669173 T>C**		**N^- (n=102)**	**N^+ (n=99)**	**PE^- (n=101)**	**PE^+ (n=103)**	**EO PE^- (n=50)**	**EO PE^+ (n=52)**	**EO PE (n=102)**	**LO PE^- (n=51)**	**LO PE^+ (n=51)**	**LO PE (n=102)**	**N (n=201)**	**PE (n=204)**	**HIV^- (n=203)**	**HIV^+ (n=202)**
Genotype	TT	40 (39.22%)	33 (33.33%)	28 (27.72%)	30 (29.13%)	12 (24.00%)	18 (34.62%)	30 (29.41)	16 (31.37%)	12 (23.53%)	28 (27.45)	73 (36.32%)	58 (28.43%)	68 (33.50%)	63 (31.19%)
	TC	45 (44.12%)	22 (22.22%)	45 (44.55%)	45 (43.69%)	22 (44.00%)	22 (42.31%)	44 (43.14)	23 (45.10%)	23 (45.10%)	46 (45.10)	87 (43.28%)	90 (44.12%)	90 (44.33%)	87 (43.07%)
	CC	17 (16.67%)	24 (24.24%)	28 (27.72%)	28 (27.18%)	16 (32.00%)	12 (23.08%)	28 (27.45)	12 (23.53%)	16 (31.37%)	28 (27.45)	41 (20.40%)	56 (27.45%)	45 (22.17%)	52 (25.74%)
Allele	T	125 (61.27%)	108 (54.55%)	101 (50.00%)	105 (50.97%)	46 (46.00%)	58 (55.77%)	104 (50.98)	55 (53.92%)	47 (46.08%)	102 (50.00)	233 (57.96%)	206 (50.49%)	226 (55.67%)	213 (52.72%)
	C	79 (37.73%)	90 (45.45%)	101 (50.00%)	101 (49.03%)	54 (54.00%)	46 (44.23%)	100 (49.02)	47 (46.08%)	55 (53.92%)	102 (50.00)	169 (42.04%)	202 (49.51%)	180 (44.33%)	191 (47.28%)
**rs7521189 G>A**		**N^- (n=102)**	**N^+ (n=99)**	**PE^- (n=101)**	**PE^+ (n=103)**	**EO PE^- (n=50)**	**EO PE^+ (n=52)**	**EO PE (n=102)**	**LO PE^- (n=51)**	**LO PE^+ (n=51)**	**LO PE (n=102)**	**N (N=201)**	**PE (n=204)**	**HIV^- (n=203)**	**HIV^+ (n=202)**
Genotype	GG	28 (27.45%)	27 (27.27%)	30 (29.70%)	37 (35.92%)	16 (32.00%)	14 (26.92%)	30 (29.41)	14 (27.45%)	23 (45.10%)	37 (36.27)	55 (27.36%)	67 (32.84%)	58 (28.57%)	64 (31.68%)
	GA	42 (41.18%)	47 (47.47%)	51 (50.50%)	48 (46.60%)	24 (48.00%)	28 (53.85%)	52 (50.98)	27 (52.94%)	20 (39.22%)	47 (46.08)	89 (44.28%)	99 (48.53%)	93 (45.81%)	95 (47.03%)
	AA	32 (31.37%)	25 (25.25%)	20 (19.80%)	18 (17.48%)	10 (20.00%)	10 (19.23%)	20 (19.61)	10 (19.61%)	8 (15.69%)	18 (17.65)	57 (28.36%)	38 (18.63%)	52 (25.62%)	43 (21.29%)
Allele	G	98 (48.04%)	101 (51.01%)	111 (54.95%)	122 (59.22%)	56 (56.00%)	56 (53.85%)	112 (54.90)	55 (53.92%)	66 (64.71%)	121 (59.31)	199 (49.50%)	233 (57.11%)	209 (51.48%)	223 (55.20%)
	A	106	97	91	84 (40.78%)	44	48	92 (45.10)	47	36	83 (40.69)	203	175	197	181

		(51.96%)	(48.99%)	(45.05%)		(44.00%)	(46.15%)		(46.08%)	(35.29%)		(50.50%)	(42.89%)	(48.52%)	(44.80%)
**rs805305 C>G**		**N^- (n=102)**	**N^+ (n=99)**	**PE^- (n=101)**	**PE^+ (n=103)**	**EO PE^- (n=50)**	**EO PE^+ (n=52)**	**EO PE (n=102)**	**LO PE^- (n=51)**	**LO PE^+ (n=51)**	**LO PE (n=102)**	**N (N=201)**	**PE (n=204)**	**HI V^- (n=203)**	**HI V^+ (n=202)**
Genotype	GG	6 (5.88%)	9 (9.09%)	13 (12.87%)	10 (9.71%)	9 (18.00%)	5 (9.62%)	14 (13.73)	4 (7.84%)	5 (9.80%)	9 (8.82)	15 (7.46%)	23 (11.27%)	19 (9.36%)	19 (9.41%)
	CG	23 (22.55%)	33 (33.33%)	22 (21.78%)	28 (27.18%)	9 (18.00%)	12 (23.08%)	21 (20.59)	13 (25.49%)	16 (31.37%)	29 (28.43)	56 (27.86%)	50 (24.51%)	45 (22.17%)	61 (30.20%)
	CC	73 (71.57%)	57 (57.58%)	66 (65.35%)	65 (63.11%)	32 (64.00%)	35 (67.31%)	67 (65.69)	34 (66.67%)	30 (58.82%)	64 (62.75)	130 (64.68%)	131 (64.22%)	139 (68.47%)	122 (60.40%)
Allele	G	35 (17.16%)	51 (25.76%)	48 (23.76%)	48 (23.30%)	27 (27.00%)	22 (21.15%)	49 (24.02)	21 (20.59%)	26 (25.49%)	47 (23.04)	86 (21.39%)	96 (23.53%)	83 (20.44%)	99 (24.50%)
	C	169 (82.84%)	147 (74.24%)	154 (76.24%)	158 (76.70%)	73 (73.00%)	82 (78.85%)	155 (75.98)	81 (79.41%)	76 (74.51%)	157 (76.96)	316 (78.61%)	312 (76.47%)	323 (79.56%)	305 (75.50%)
**rs3131383 G>T**		**N^- (n=102)**	**N^+ (n=99)**	**PE^- (n=101)**	**PE^+ (n=103)**	**EO PE^- (n=50)**	**EO PE^+ (n=52)**	**EO PE (n=102)**	**LO PE^- (n=51)**	**LO PE^+ (n=51)**	**LO PE (n=102)**	**N (N=201)**	**PE (n=204)**	**HI V^- (n=203)**	**HI V^+ (n=202)**
Genotype	GG	71 (69.61%)	66 (66.67%)	54 (53.47%)	65 (63.11%)	30 (60.00%)	38 (73.08%)	68 (66.67)	24 (47.06%)	27 (52.94%)	51 (50.00)	137 (68.16%)	119 (58.33%)	125 (61.58%)	131 (64.85%)
	GT	16 (15.69%)	20 (20.20%)	26 (25.74%)	22 (21.36%)	12 (24.00%)	9 (17.31%)	21 (20.59)	14 (27.45%)	13 (25.49%)	27 (26.47)	36 (17.91%)	48 (23.53%)	42 (20.69%)	42 (20.79%)
	TT	15 (14.71%)	13 (13.13%)	21 (20.79%)	16 (15.53%)	8 (16.00%)	5 (9.62%)	13 (12.75)	13 (25.49%)	11 (21.57%)	24 (23.53)	28 (13.93%)	37 (18.14%)	36 (17.73%)	29 (14.36%)
Allele	G	158 (77.45%)	152 (76.77%)	134 (66.34%)	152 (73.79%)	72 (72.00%)	85 (81.73%)	157 (76.96)	62 (60.78%)	67 (65.69%)	129 (63.24)	310 (77.11%)	286 (70.10%)	292 (71.92%)	304 (75.25%)
	T	46 (22.55%)	46 (23.23%)	68 (33.66%)	54 (26.21%)	28 (28.00%)	19 (18.27%)	47 (23.04)	40 (39.22%)	35 (34.31%)	75 (36.76)	92 (22.89%)	122 (29.90%)	114 (28.08%)	100 (24.75%)

N, normotensive; N-, normotensive HIV-negative; N^{+}, normotensive HIV-positive; PE, preeclampsia; $EOPE^{-}$, early onset preeclampsia HIV-negative; $EOPE^{+}$, early onset preeclampsia HIV-positive; $LOPE^{-}$, late onset preeclampsia HIV-negative; $LOPE^{+}$, late onset preeclampsia HIV-positive; HIV^{-}, HIV-negative; HIV^{+}, HIV-positive. Genotypic and allelic frequency distribution is represented as a percentage (%).
